# NanoAbLLaMA: construction of nanobody libraries with protein large language models

**DOI:** 10.3389/fchem.2025.1545136

**Published:** 2025-02-25

**Authors:** Xin Wang, Haotian Chen, Bo Chen, Lixin Liang, Fengcheng Mei, Bingding Huang

**Affiliations:** ^1^ College of Big Data and Internet, Shenzhen Technology University, Shenzhen, China; ^2^ Chengdu NBbiolab. CO., LTD., SME Incubation Park, Chengdu, China

**Keywords:** reinforcement learning, generative AI, nanobodies, libraries, protein large language models

## Abstract

**Introduction:**

Traditional methods for constructing synthetic nanobody libraries are labor-intensive and time-consuming. This study introduces a novel approach leveraging protein large language models (LLMs) to generate germline-specific nanobody sequences, enabling efficient library construction through statistical analysis.

**Methods:**

We developed NanoAbLLaMA, a protein LLM based on LLaMA2, fine-tuned using low-rank adaptation (LoRA) on 120,000 curated nanobody sequences. The model generates sequences conditioned on germlines (IGHV3-301 and IGHV3S5301). Training involved dataset preparation from SAbDab and experimental data, alignment with IMGT germline references, and structural validation using ImmuneBuilder and Foldseek.

**Results:**

NanoAbLLaMA achieved near-perfect germline generation accuracy (100% for IGHV3-301, 95.5% for IGHV3S5301). Structural evaluations demonstrated superior predicted Local Distance Difference Test (pLDDT) scores (90.32 ± 10.13) compared to IgLM (87.36 ± 11.17), with comparable TM-scores. Generated sequences exhibited fewer high-risk post-translational modification sites than IgLM. Statistical analysis of CDR regions confirmed diversity, particularly in CDR3, enabling the creation of synthetic libraries with high humanization (>99.9%) and low risk.

**Discussion:**

This work establishes a paradigm shift in nanobody library construction by integrating LLMs, significantly reducing time and resource demands. While NanoAbLLaMA excels in germline-specific generation, limitations include restricted germline coverage and framework flexibility. Future efforts should expand germline diversity and incorporate druggability metrics for clinical relevance. The model’s code, data, and resources are publicly available to facilitate broader adoption.

## 1 Introduction

Nanobodies, derived from the heavy-chain antibodies of camelids, are single-domain antibody fragments that lack light chains (Hamers-Casterman et al., 1993). They possess unique characteristics, such as high stability and ease of production, which are of significant importance for diagnostics, therapeutics, and molecular research (Mullin et al., 2024). The antigen-binding site of nanobodies is composed of three complementarity-determining regions (CDR1, CDR2, and CDR3), which play a crucial role in antigen recognition and binding. CDR1 and CDR2 are relatively short and primarily provide auxiliary functions for antigen binding, while CDR3 is longer and highly diverse, serving as the key region for antigen binding. The rapid development of nanobodies highlights the importance of constructing diverse and high-quality nanobody libraries (Kunz et al., 2018).

Traditional methods for preparing synthetic nanobody libraries typically use a defined protein framework as a template, with artificial rules for designing the CDR3 region sequences. Although effective, these methods are usually time-consuming and restrictive, limiting the speed of discovery and development (Valdés-Tresanco et al., 2022). Nowadays, there are already some synthetic binding protein databases, such as the SYNBIP database. The nanobody libraries generated in this database are also potential synthetic protein binders (Li et al., 2024). With the continuous breakthroughs in the field of deep learning, especially the emergence of protein large language models (ProLLM), a new approach has been provided for the construction of nanobody libraries (Strokach and Kim, 2022).

Protein large language models, after training on a vast dataset of protein sequences and structural data, have the capability to generate new protein sequences with desired characteristics. These models use deep learning techniques to accurately understand protein sequences and predict protein folding to generate required protein sequences (Varadi et al., 2021; Ofer et al., 2021). By integrating these models into the construction process of nanobody libraries, researchers can significantly improve the efficiency, diversity, and specificity of library generation.

This paper explores the innovative application of protein large language models in constructing nanobody libraries. We trained the model on a nanobody dataset and evaluated its effectiveness in generating a diverse range of nanobody sequences. The model is capable of generating the required nanobody sequences conditioned on different germlines. By leveraging the predictive power of protein large language models, we aim to provide a new method for the preparation of nanobody libraries.

## 2 Methods

### 2.1 Nanobody sequence dataset

In the training of NanoAbLLaMA, we utilized ProLLaMA as the underlying model framework. ProLLaMA is a large language model for proteins that has been pre-trained on the LLaMA2 framework, specializing in protein language. Its superior scalability allows for the utilization of natural language to formulate user instructions and to create our dataset (Lv et al., 2024). We extracted nanobody sequences from SAbDab database and experimental datasets (Dunbar et al., 2013). The experimental dataset is the base sequence, since the final goal is to create a synthetic nanobody library for germlines IGHV3- $3^{*}$ 01 and IGHV3S $53^{*}$ 01, we selected sequences for germlines IGHV3- $3^{*}$ 01 and IGHV3S $53^{*}$ 01 after correct codon frame translation. After removing sequences with obviously unreasonable lengths, we were left with 260,000 unique nanobody sequences. Then, we aligned all sequence frameworks with the germline V genes of the alpaca antibody in the IMGT database (Lefranc et al., 1998; Lefranc, 2008) leaving sequences with more than 85% consistency, totaling 120,000. Finally, we allocated 80% of the data to the training set and 20% to the test set.

### 2.2 Model architecture and training framework

Large Language Model Meta AI 2 (LLaMA2) is the second-generation large language model developed by Meta, based on the classic Transformer architecture (Touvron et al., 2023). We train on the LLaMA2 framework using Low-Rank Adaptation (LoRA) (Hu et al., 2021) to reduce training costs. LoRA is an efficient method for fine-tuning large pre-trained models, aiming to reduce the computational resources and storage space required for fine-tuning. LoRA introduces low-rank matrix decomposition technology, decomposing the model’s weight matrix into two low-rank matrices, thereby greatly reducing the number of parameters that need to be updated. Conceptually, fine-tuning can be thought of as a process of finding parameter changes (Ding et al., 2023). Let W ∈R ^d×k^ be a weight matrix in a pre-trained model, where d and k represent the dimensions of input and output, respectively. LoRA decomposes the weight matrix W into two low-rank matrices A ∈R ^d×r^ and B ∈R ^r×k^, where r ≪ d,k.

$$W=W_{0}+A\times B,$$ (1)

Here, $W_{0}$ is the fixed weight matrix during pre-training, and A and B are the low-rank matrices that need to be learned during fine-tuning. By such decomposition, only A and B need to be updated during fine-tuning without changing $W_{0}$ . A and B can be integrated into the original model using Equation 1. Finally, LoRA can prevent catastrophic forgetting of the original knowledge because the rank of the newly learned knowledge is lower than that of the original knowledge (Aghajanyan et al., 2020).

**FIGURE 1:**
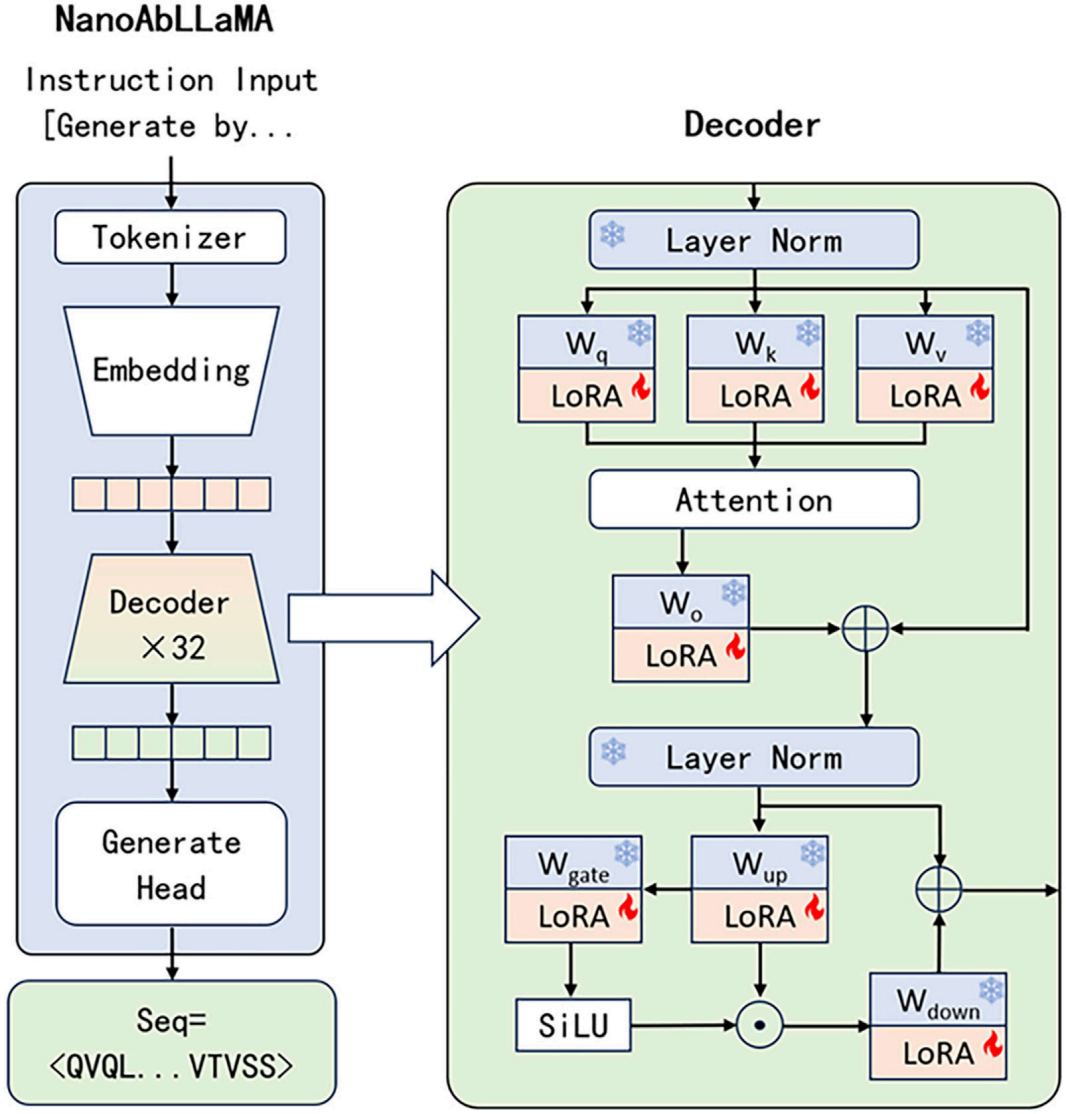
The overall architecture of NanoAbLLaMA. We added low-rank adapters (LoRA) to certain weights. During training, we freeze these weights and other parameters, focusing only on training LoRA.

**FIGURE 2:**
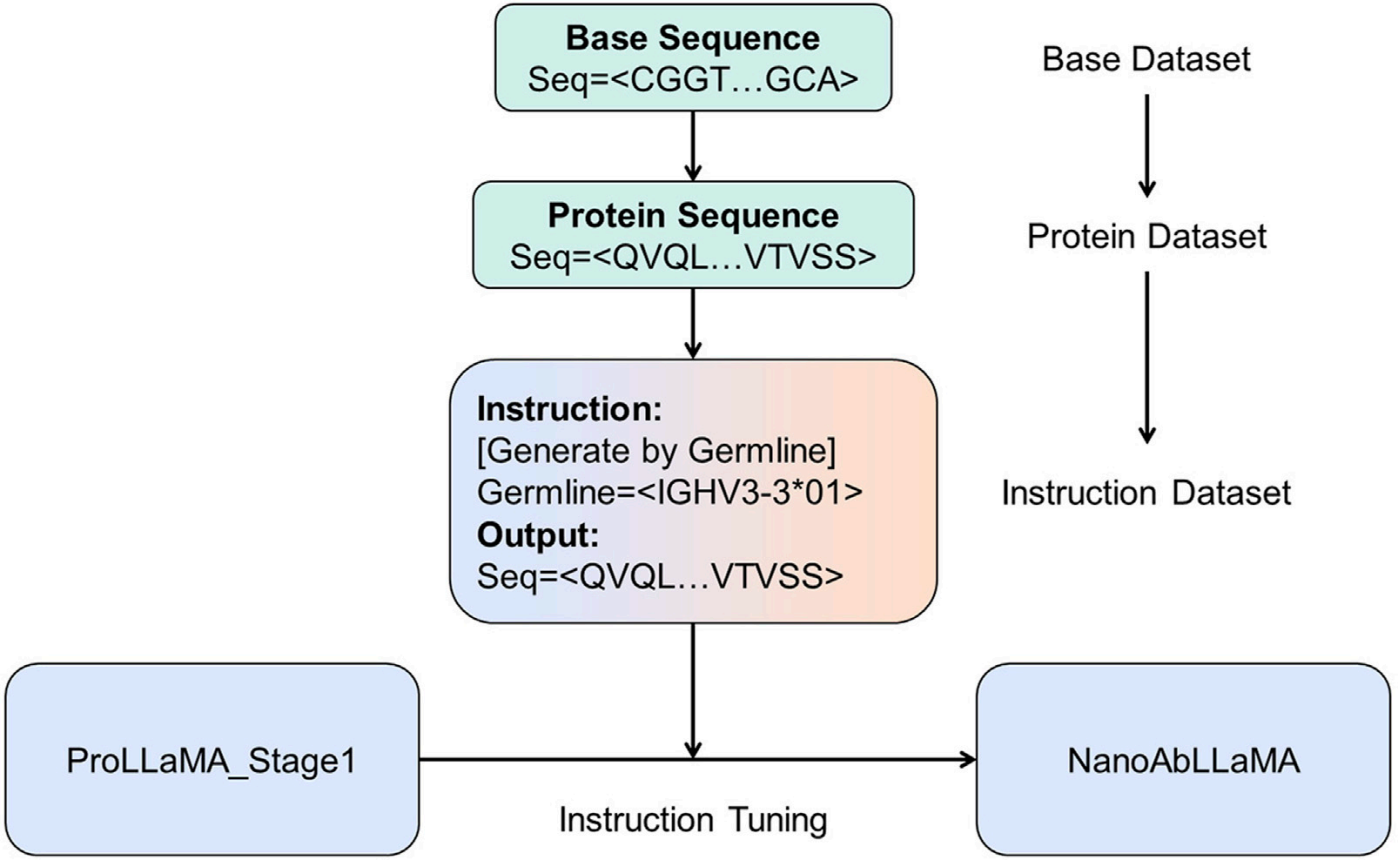
The training process of NanoAbLLaMA. The base data is translated from our own base data to obtain protein sequences, which are then made into instruction datasets for training.

We add LoRA to every decoder in LLaMA2, including $w_{q},w_{k},w_{v},w_{o},w_{\mathrm{gate}},w_{up}$ and $w_{\mathrm{down}}$ . The original parameters of LLaMA2 will be frozen, and only LoRA can be trained. Benefiting from LoRA, we effectively reduced the number of parameters that needed to be trained in the model, and also significantly reduced the training cost, so that we only trained about 6% of the parameters. The model architecture is shown in Figure 1, and the training process diagram is shown in Figure 2.

### 2.3 Synthetic library

Aim for a synthetic library with high humanization and fewer risk sites, we independently generate CDR1, CDR2, and CDR3. To create a synthetic library, we need to generate a certain amount of data for statistical analysis and then create a synthetic library based on the statistical patterns. To ensure that the synthetic library can be applied in practice, we use frameworks with high humanization and fewer risk sites to generate our data. We obtained the required frameworks from the SAbDab database (Schneider et al., 2021). For example, in the germline IGHV3- $3^{*}$ 01, the sequence we chose is:

EVQLVESGGGLVQPGGSLRLSCAASGRTFSYNPMGWFR QAPGKGRELVAAISRTGGSTYYPDSVEGRFTISRDNAKRM VYLQMNSLRAEDTAVYYCAAAGVRAEDGRVRTLPSEYTF WGQGTQVTVSS

And in the germline IGHV3S $53^{*}$ 01, the sequence we chose is: EVQLLESGGGEVQPGGSLRLSCAASGFSFSINAMGWYRQAP GKRREFVAAIESGRNTVYAESVKGRFTISRDNAKNTVYLQ MSSLRAEDTAVYYCGLLKGNRVVSPSVAYWGQGTLVTVKP

These two frameworks have a humanization rate as high as 99.9%, with fewer than 10 risk sites, making them very suitable for use as frameworks for generation. We generated 10,000 nanobody sequences for CDR1 and CDR2 regions using these two frameworks. Due to the lower diversity in CDR1 and CDR2 regions, the overall repetition rate is relatively high, but this also ensures the correctness of the statistical results. However, due to the higher diversity in the CDR3 region, we generated a total of 100,000 unique nanobody sequences. To ensure the correctness of the statistical results, for the germline IGHV3- $3^{*}$ 01, we selected data with CDR3 lengths from 15 to 19 for statistics, and for the germline IGHV3S $53^{*}$ 01, we selected data with CDR3 lengths from 12 to 16 for statistics because they have the most data.

## 3 Results

### 3.1 NanoAbLLaMA

Our model NanoAbLLaMA was trained on 120,000 nanobody sequences for full-length nanobody sequence generation. NanoAbLLaMA can generate sequences conditioned on germline (IGHV3- $3^{*}$ 01 or IGHV3S $53^{*}$ 01).

### 3.2 Controllable germline generation

To verify that the results generated by our model are valid, we generated 10,000 unique sequences for each of the two germlines (IGHV3- $3^{*}$ 01 and IGHV3S $53^{*}$ 01), then identified them in AbNumber (Dunbar and Deane, 2016), specified the numbering method and species, and judged the germline generation accuracy.

The results shown in Table 1 indicate that NanoAbLLaMA can generate corresponding nanobody sequences based on instructions for the required germline, thus achieving controllable germline generation.

**TABLE 1:** Germline generation accuracy. Our NanoAbLLaMA achieved nearly 100% accuracy for the generation of both germlines.

	IGHV3-3*01	IGHV3S53*01
Accuracy	100.0%	95.5%

### 3.3 Nanobody sequence generation

**FIGURE 3:**
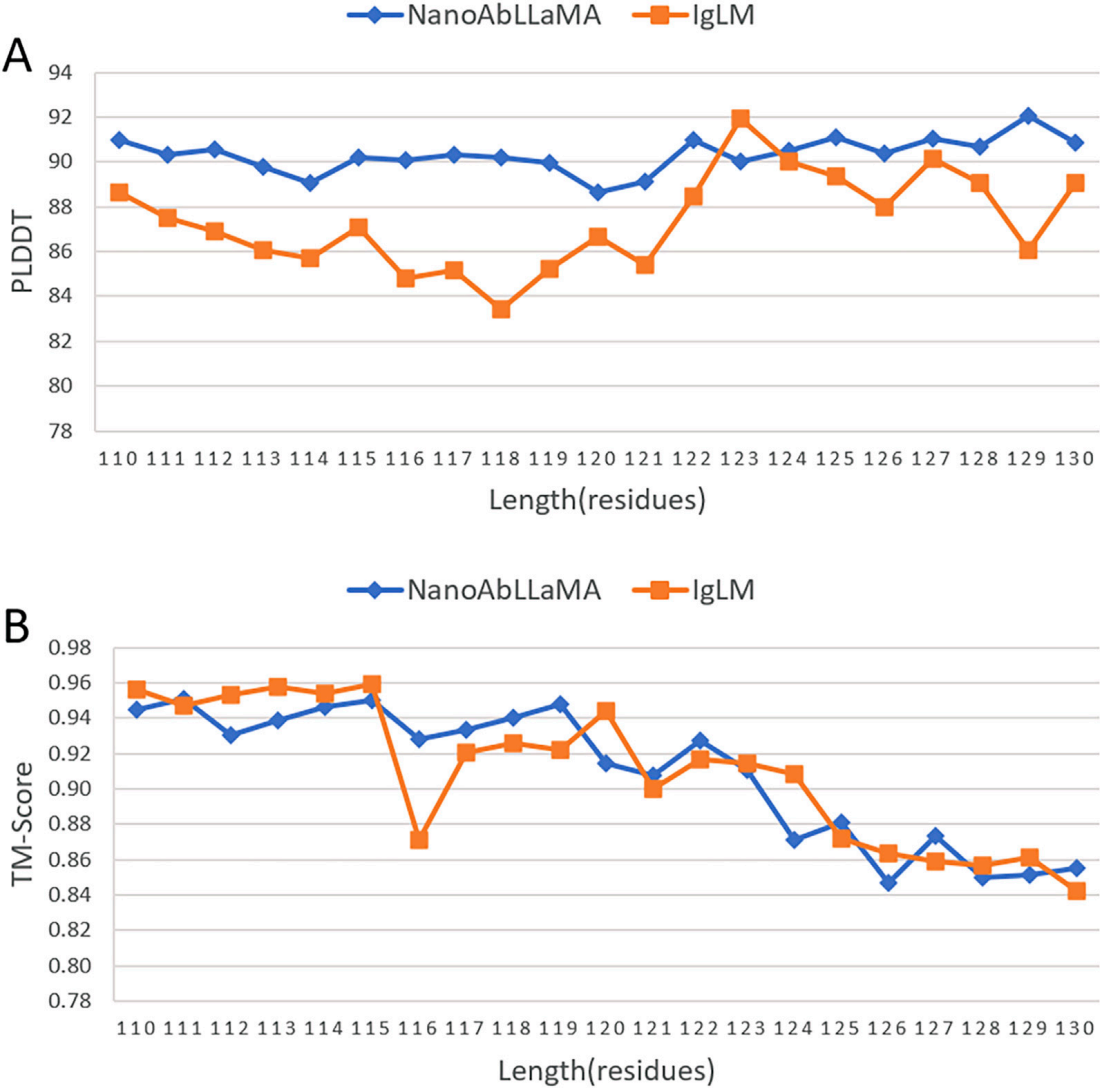
Structural experiments of NanoAbLLaMA. **(A)** Compared with IgLM, NanoAbLLaMA has a higher pLDDT score. **(B)** The TM-Score score is comparable to IgLM.

**FIGURE 4:**
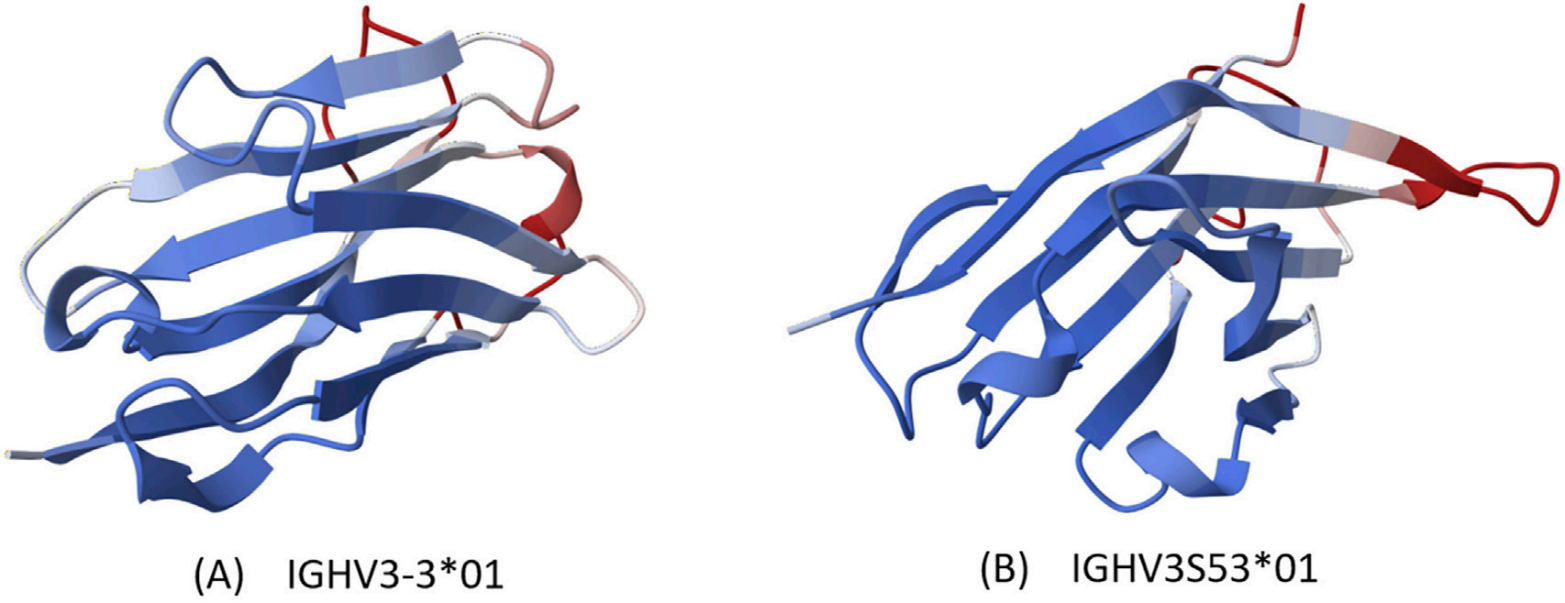
Protein visualization. Structures generated by AlphaFold based on sequences, with color representing credibility, blue being more credible. **(A)** IGHV3-3*01 **(B)** IGHV3S53*01.

We compared our model with other state-of-the-art models in the field of antibody design, such as the Immunoglobulin Language Model (IgLM) (Shuai et al., 2023), which is a generative language model that uses bidirectional context to design antibody sequence spans of different lengths and is trained on a large-scale natural antibody dataset. IgLM can generate full-length antibody sequences conditioned on chain type and source species. To ensure the correctness of the calculation results, we selected the most abundant data for statistics, that is, sequences with lengths of 110 aa to 130 aa, and selected 10 sequences of each length for statistical averaging. We used ImmuneBuilder (Abanades et al., 2023) to predict the structure of the sequences. ImmuneBuilder is a set of deep learning models specifically for predicting the structures of antibodies, nanobodies, and T cell receptors, which is highly accurate and much faster than AlphaFold2. We calculated the predicted Local Distance Difference Test (pLDDT) (Varadi et al., 2021) based on the predicted structure, and pLDDT is used to measure whether the sequence is structurally reasonable. At the same time, we used Foldseek (van Kempen et al., 2024) to calculate the average template modeling score (TM-Score) (Zhang and Skolnick, 2004) and the root mean square deviation (RMSD), which reflect the degree of structural similarity. TM-score focuses more on the overall structure, while RMSD is more sensitive to the size and local changes of protein structures. The results are shown in Figure 3, our NanoAbLLaMA has a better pLDDT score, indicating that NanoAbLLaMA, through training on nanobody sequence data, can produce structurally reasonable nanobody sequences. The average value and standard deviation of pLDDT for the nanobody sequences generated by NanoAbLLaMA are 90.32 ± 10.13, while those for IgLM are 87.36 ± 11.17 Figure 4 displays the 3D structure of the generated sequences, and Table 2 shows the structural scores.

**TABLE 2:** Structural scoring table.

	pLDDT	TM-score	RMSD
NanoAbLLaMA	90.32 ± 10.13	0.91 ± 0.04	1.43 ± 0.26
IgLM	87.36 ± 11.17	0.91 ± 0.04	1.24 ± 0.30

**FIGURE 5:**
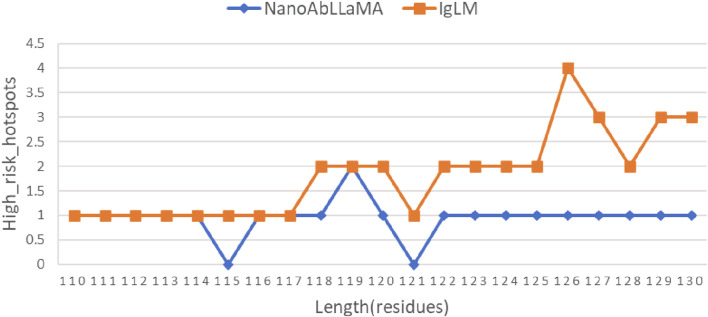
Comparison of the number of high-risk sites. The number of high-risk sites in the first half is quite similar, and the number of high-risk sites in our model is lower in the second half.

At the same time, the production of nanobody synthetic libraries also pays attention to post-translational modifications (PTM) (Ramazi and Zahiri, 2021) of proteins, which are common risks in biopharmaceutical development. Mainly including: Oxidation, Glycosylation, Hydrolysis, etc. Therefore, corresponding detection was also carried out. The results are shown in Figure 5, the number of high-risk sites in the sequences generated by our NanoAbLLaMA is less than that of IgLM.

**FIGURE 6:**
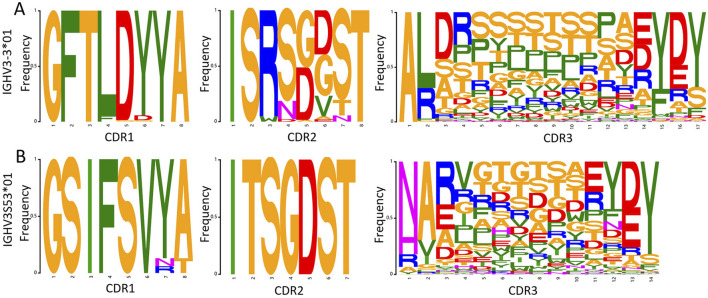
Amino acid frequency of each CDR region for the two germlines. **(A)** Amino acid frequency for germline IGHV3- $3^{*}$ 01, only data with a length of 17 for CDR3 is shown, it can be seen that the sequences in CDR1 and CDR2 regions are relatively stable, while the changes in CDR3 region are larger. **(B)** Amino acid frequency for germline IGHV3S $53^{*}$ 01, only data with a length of 14 for CDR3 is shown, it can be seen that the sequence length is shorter.

After generating sequences with NanoAbLLaMA, we still need to make a synthetic library, so we need to statistically determine the frequency of each amino acid site and make a synthetic library based on germline, framework, and amino acid frequency. Figure 6 is a SeqLogo created based on a portion of the data we generated.

We utilized Discovery Studio to analyze the disulfide bond information of the generated sequences. The analysis revealed that all sequences contain a conserved disulfide bond connecting FR1 (C23) and FR3 (C104), which plays a crucial role in maintaining the structural stability of the protein. This conserved disulfide bond is a common feature in many proteins, contributing to their overall stability and functionality. However, no additional disulfide bonds were identified in the sequences.

## 4 Discussion

In this study, we propose an innovative approach to construct nanobody synthesis libraries using the nanobody large language model NanoAbLLaMA. This method not only improves the efficiency of library construction, but also provides new ideas for the design of nanobodies. The following is an in-depth discussion of our findings.

First of all, traditional nanobody library construction methods often rely on multi-step operations in the laboratory, involving many complex steps such as single-stranded antibody collection, vector preparation, and insertion of single-stranded antibody sequences. These methods are time-consuming and costly, limiting the widespread use of nanobodies. Our NanoAbLLaMA model leverages the powerful generation capabilities of large language models to rapidly generate high-quality nanobody sequences and generate libraries based on statistical analysis, significantly reducing the time and resource consumption of library construction.

Secondly, the existing antibody language models, such as AbLang (Olsen et al., 2022) and IgLM(Shuai et al., 2023), cannot meet our requirements for generating nanobody sequences based on germline, and most of these models use species and chain type as conditions to generate sequences. The NanoAbLLaMA model is trained using a low-rank adaptive technique, which allows it to be fine-tuned for specific germlines. The effectiveness of this strategy was validated in our experiments, where the model was able to generate the expected nanoantibody sequences and excelled in diversity and specificity. These results indicate that the combination of the flexibility of large language models and the advantages of targeted training can effectively improve the design efficiency and quality of nanobodies.

However, there are some limitations to this study. First, although NanoAbLLaMA has achieved good results in generating germline-specific nanoantibody sequences, it is unable to cover more germlines and cannot specify the framework to generate CDR sequences. Future research may consider expanding the training dataset to cover a wider range of germlines, or modifying the training mode of the model to improve the model’s capabilities.

In addition, factors such as druggability still need to be paid attention to in practical application. These factors are critical for the clinical application of nanobodies and therefore need to be explored in depth in follow-up studies.

## 5 Conclusion

Existing methods for making nanobody synthetic libraries are mature but laborious. In this work, we introduced a new way to make nanobody synthetic libraries by generating nanobody sequences with protein large language models and making nanobody synthetic libraries based on statistical results. We also developed NanoAbLLaMA, a ProLLM that can generate nanobody sequences based on germline. Experiments show that NanoAbLLaMA has excellent performance.

## Data Availability

The original contributions presented in the study are included in the article/Supplementary Material, further inquiries can be directed to the corresponding authors.
