# Supplementary material for: NanoAbLLaMA: construction of nanobody libraries with protein large language models
Source: Front Chem. 2025 Feb 25;13:1545136. doi: 10.3389/fchem.2025.1545136 (PMC11893428; doi:10.3389/fchem.2025.1545136)
Supplement: Supplementary file 1 [file DataSheet1.pdf]

Supplementary Material

1 DETAILED TRAINING AND GENERATION SETTINGS

Training settings: We used LoRA for optimization, with LoRA rank set to 64, alpha set to 128, and dropout set to 0.05. Model parameters include the use of AdamW as the optimizer, the scheduler type is cosine, the peak learning rate is 0.05, with two training epochs. The maximum sequence length is set to 256, and the gradient accumulation steps are 4. The warm-up ratio is 0.03, and weight decay is set to 0.01. The data type is bfloat16. The batch size per GPU is 36.

Generation settings: The temperature is set to 0.4, top_k is set to 40, top_p is set to 0.95, do_sample is set to True, num_beams are set to 1, repetition_penalty is set to 1.2, and max_new_tokens are set to 400.

2 SUPPLEMENTARY FIGURES

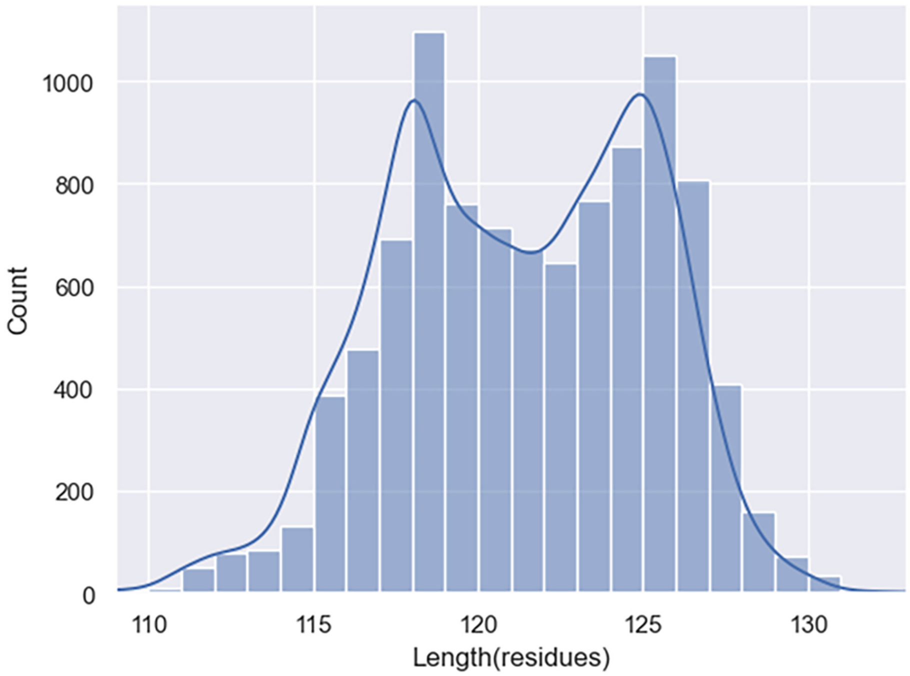

Figure S1. Generated sequence lengths.

Most generated sequence lengths fall between 110 aa and 130 aa, which is consistent with the typical length of nanobody sequences.

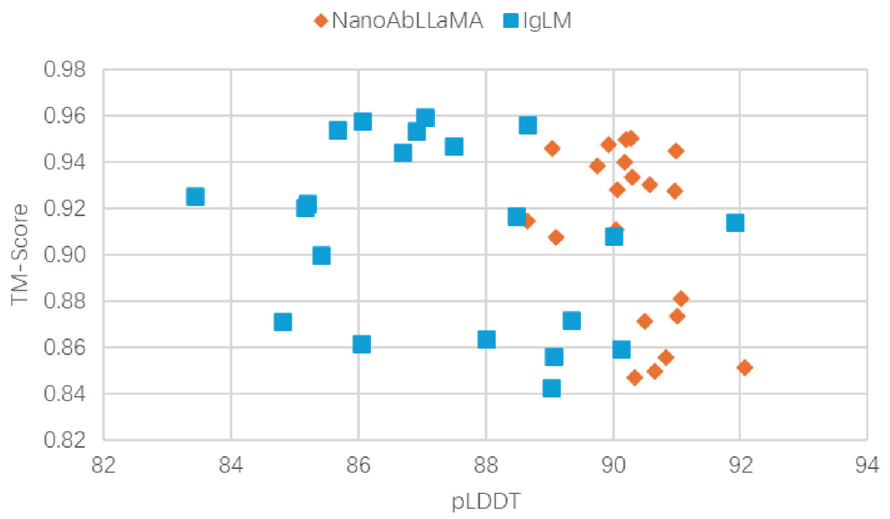

Figure S2. Distribution of pLDDT and TM-Score, where one spot denotes one sequence.

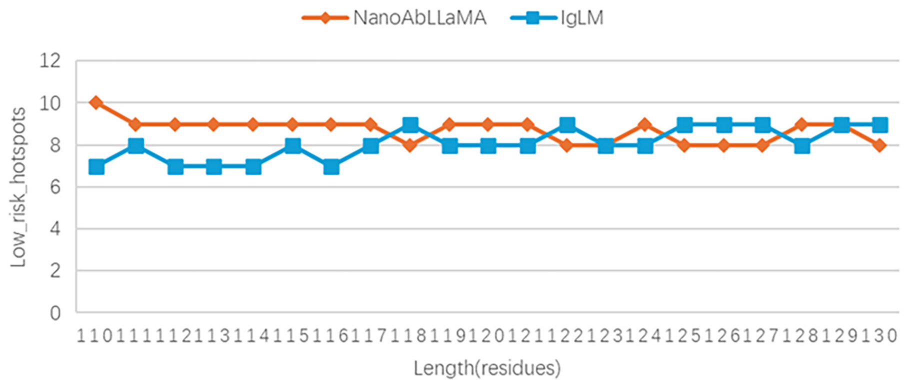

Figure S3. Comparison of the number of low-risk sites, both numbers are similar.
